# *NPM1* alternative transcripts are upregulated in acute myeloid and lymphoblastic leukemia and their expression level affects patient outcome

**DOI:** 10.1186/s12967-018-1608-2

**Published:** 2018-08-20

**Authors:** Luiza Handschuh, Pawel Wojciechowski, Maciej Kazmierczak, Malgorzata Marcinkowska-Swojak, Magdalena Luczak, Krzysztof Lewandowski, Mieczyslaw Komarnicki, Jacek Blazewicz, Marek Figlerowicz, Piotr Kozlowski

**Affiliations:** 1 European Centre for Bioinformatics and Genomics, Institute of Bioorganic Chemistry, Polish Academy of Sciences, Noskowskiego 12/14, 61-704 Poznan, Poland; 2 Department of Hematology and Bone Marrow Transplantation, Poznan University of Medical Sciences, Szamarzewskiego 84, 60-569 Poznan, Poland; 3 Institute of Technology and Chemical Engineering, Poznan University of Technology, Poznan, Poland; 4 Institute of Computing Science, Poznan University of Technology, Piotrowo 2, 60-965 Poznan, Poland

**Keywords:** *NPM1*, Expression, AML, ALL, Splice variants, Quantitative transcript analysis

## Abstract

**Background:**

Expression of the *NPM1* gene, encoding nucleophosmin, is upregulated in cancers. Although more than ten *NPM1* transcripts are known, the reports were usually limited to one predominant transcript. In leukemia, the *NPM1* expression has not been widely studied so far. In acute myeloid leukemia (AML), the mutational status of the gene seems to play a pivotal role in carcinogenesis. Therefore, the aim of the study was to quantify alternative *NPM1* transcripts in two types of acute leukemia, AML and ALL (acute lymphoblastic leukemia).

**Methods:**

Using droplet digital PCR, we analyzed the levels of three protein-coding *NPM1* transcripts in 66 samples collected from AML and ALL patients and 16 control samples. Using RNA-seq, we detected 8 additional *NPM1* transcripts, including non-coding splice variants with retained introns. For data analysis, Welch two sample t-test, Pearson’s correlation and Kaplan–Meier analysis were applied.

**Results:**

The levels of the particular *NPM1* transcripts were significantly different but highly correlated with each other in both leukemia and control samples. Transcript *NPM1.1*, encoding the longest protein (294 aa), had the highest level of accumulation and was one of the most abundant transcripts in the cell. Comparing to *NPM1.1,* the levels of the *NPM1.2* and *NPM1.3* transcripts, encoding a 265-aa and 259-aa proteins, were 30 and 3 times lower, respectively. All three *NPM1* transcripts were proportionally upregulated in both types of leukemia compared to control samples. In AML, the levels of *NPM1* transcripts decreased in complete remission and increased again with relapse of the disease. Low levels of *NPM1.1* and *NPM1.3* were associated with better prognosis. The contribution of non-coding transcripts to the total level of *NPM1* gene seemed to be marginal, except for one short 5-end transcript accumulated at high levels in AML and control cells. Aberrant proportions of particular *NPM1* splice variants could be linked to abnormal expression of genes encoding alternative splicing factors.

**Conclusions:**

The levels of the studied *NPM1* transcripts were different but highly correlated with each other. Their upregulation in AML and ALL, decrease after therapy and association with patient outcome suggests the involvement of elevated *NPM1* expression in the acute leukemia pathogenesis.

**Electronic supplementary material:**

The online version of this article (10.1186/s12967-018-1608-2) contains supplementary material, which is available to authorized users.

## Background

Nucleophosmin (NPM), also known as nucleolar phosphoprotein B23, nucleolar protein NO38 or numatrin, is involved in a wide spectrum of essential cell processes, including ribosome biogenesis and export, control of centrosome duplication, protein chaperoning, histone and nucleosome assembly, cell proliferation, DNA repair and regulation of genome stability through interaction with tumor suppressors p53 and ARF (alternative reading frame) [1, 2]. The protein constantly moves between the nucleus and the cytoplasm, but its major localization is the nucleolus where ribosome assembly occurs [3].

**Fig. 1:**
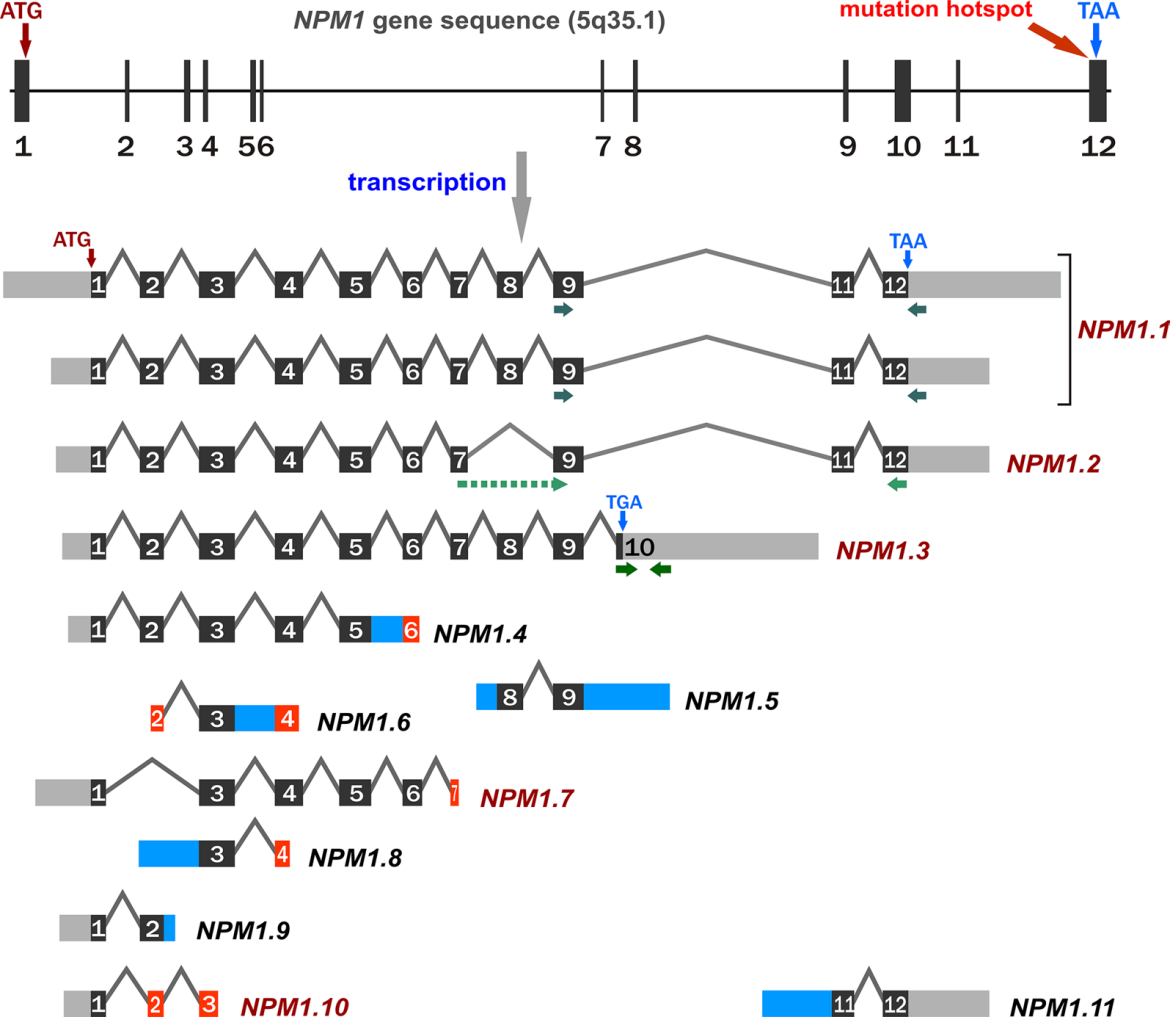
The schematic presentation of *NPM1* gene structure and transcript variants. Exons are represented by numbered blocks. The black color marks the protein coding sequence. The red color indicates exons that are included in transcripts as partial sequences, lacking the 5′ or 3′ fragments of the exons present in the *NPM1.1* transcript variant. The names of protein coding transcripts are written in dark red whereas the names of non-coding variants with retained introns/intron fragments (blue blocks) are written in black. The green arrows under transcripts indicate ddPCR primer positions. One of the *NPM1.2*-specific primers crosses the exon boundary (indicated by the dotted line linking exons 7 and 9)

The ubiquitously expressed and evolutionarily conserved gene encoding NPM, *NPM1* (Gene ID 4869, mapped at 5q35.1, genomic NCBI Reference Sequence NG_016018.1, Ensembl ENSG00000181163), is mutated in 25–35% of adult patients with primary acute myeloid leukemia (AML) and 46–64% of adult patients with normal karyotype AML (NK-AML) [3–5]. A mutation, usually an out-of-frame tetranucleotide insertion in the last (12th) *NPM1* exon, changes the C-terminus of the protein and causes aberrant cytoplasmic accumulation of the protein (NPMc+) [1, 3, 5]. NPMc + AML reveals unique biological and clinical features and distinct mRNA and miRNA expression profiles [6–8]. The presence of an *NPM1* mutation without concomitant *FLT3*-ITD (FMS-like tyrosine kinase 3-internal tandem duplication) in NK-AML patients has been associated with a favorable prognosis [9–11]. Therefore, the World Health Organization (WHO) has recommended distinguishing AML with an *NPM1* mutation as a separate entity [12, 13]. Numerous tests detecting *NPM1* mutations have been developed [14–18]. In other human neoplasms, mutations in the *NPM1* gene are rare. Instead, overexpression of the *NPM1* gene is frequently observed in different solid tumors, e.g., ovarian [19], prostate [20], colon [21, 22], bladder [23], thyroid [24], lung [25] and liver [26, 27] cancers. High *NPM1* expression can be treated as an early marker of proliferative activity, preceding the S-phase of the cell cycle [28]. Increased *NPM1* expression was also detected in human-derived leukemia cell lines [29]. However, to date, *NPM1* expression in leukemia patients has rarely been reported and previous studies were conducted on a protein level or gene level without distinguishing transcript variants [30]. The exception has been a recent study by Zajac et al. [31] who focused on three protein-coding *NPM1* splice variants, revealing a higher level in AML than in healthy control samples and associated a high expression of one *NPM1* splice variant with a better prognosis in NK-AML patients. Nevertheless, the knowledge about *NPM1* transcripts is still incomplete and unsystematic. In the literature, three *NPM1* splice variants are reported, usually in the context of the protein isoforms they encode, historically named B23.1, B23.2 and B23.3 [3, 31, 32]. The corresponding transcripts are called *NPM1.1*, *NPM1.2* and *NPM1.3* by some authors [2, 33] or R1, R2 and R3 by others [31]. In addition, transcripts 2 and 3 are often confused. Database exploration demonstrated that more than three *NPM1* splice variants exist, but the number, length and nomenclature of the transcripts are not consistent between databases (Additional file 1: Table S1). According to NCBI Gene repository, 8 *NPM1* transcript variants (referred to as variants 1–8) were detected in humans, including 7 protein-coding variants. The Ensembl database lists 12 transcripts (named NPM1-201–NPM1-212), including 6 protein-coding variants. Both databases assign four transcript variants to the three main proteins mentioned above. As shown in Fig. 1, none of the transcripts comprises all 12 exons located in the genomic *NPM1* sequence. The predominant protein (294 aa, 32.6 kDa) [26] is encoded by the transcript containing 11 exons, with exon 10 missing. Because there are two transcript variants encoding this protein, we refer to them both as *NPM1.1*. Another transcript, referred to here as *NPM1.2*, lacks exons 8 and 10 and encodes a 265-aa protein, without an open reading frame (ORF) shift when compared to *NPM1.1*. The transcript referred to here as *NPM1.3* lacks the last two exons (11 and 12) but maintains exon 10. It encodes a shortened protein (259 aa) with a distinct C-terminus, lacking the region containing a nucleolar localization signal (NoLS) [1].

The aim of this work was to quantify *NPM1* transcripts in two types of acute leukemia, derived from different hematopoietic cell lineages: AML and ALL (acute lymphoblastic leukemia). First of all, we wanted to check whether *NPM1* gene is upregulated in leukemia similarly as in other human neoplasms and whether *NPM1* expression depends on the mutation status of the gene. In the case of upregulation, we were going to distinguish particular transcripts to test whether *NPM1* transcripts are similarly increased in both leukemia types and which transcripts contribute the most to the change of expression. Another objective of the study was to compare *NPM1* expression levels at three time points (at first diagnosis, after treatment and relapse). We were also interested in association of *NPM1* expression with patient outcome.

## Methods

### Samples

Peripheral blood (PB) or bone marrow (BM) samples were collected from 66 adult patients with acute leukemias at the time of first diagnosis and from 16 adult healthy volunteers (HV). Additionally, we collected 9 samples from AML patients after therapy (T1 time point) and 7 samples from AML patients in relapse (T2 time point). For the time point experiment, 9 samples from AML patients at the time of first diagnosis (T0 time point) were included (Table 1, Additional file 1: Tables S2, S3). Each person provided signed informed consent for treatment and for their participation in this study. Appropriate approval was also obtained from the Bioethical Commission of the Karol Marcinkowski University of Medical Sciences. Patients were diagnosed and treated at the Department of Hematology and Bone Marrow Transplantation in the University Hospital of the Lord’s Transfiguration of the University of Medical Sciences in Poznan, Poland. Fifty-seven patients were diagnosed with AML (with the following representations of the FAB subtypes: 1 M0, 13 M1, 23 M2, 3 M3, 13 M4, and 4 M5), 8 ALL-B (acute lymphoblastic leukemia, B-cell type) and 1 patient with mixed-phenotype acute leukemia (biphenotypic acute leukemia). Standard AML therapy using cytosine arabinoside plus daunorubicin (“3 + 7”) was administered to all patients to induce complete remission (CR), which was defined according to the European Leukemia Net guidelines [34]. Mononuclear cells from the peripheral blood (PBMCs) and bone marrow (BMMCs) were separated through density gradient centrifugation (Gradisol L, Aqua-Medica, Poland) and washed 3 times with 1 × PBS (phosphate buffered saline, Ca and Mg-free, BIOMED, Poland). The cell pellet was suspended in lysis buffer from a mirVana miRNA Isolation Kit (Ambion/Thermo Fisher Scientific, Waltham, MA, USA) and immediately frozen at − 80 °C.

**Table 1:** The summarized characteristics of patients

Disease	FAB type	Number of patients (F/M)	Age median (range)	WBC mean (range)	*NPM1*mut.	*FLT3*mut.	*RUNX1/RUNX1T1*	Karyotype
ddPCR-based analysis of *NPM1* transcripts at the time of diagnosis								
AML	M0	1 (0/1)	64	9.7	–	–	–	NA (1)
	M1	13 (7/6)	51 (25–65)	94.8 (1–310.9)	5	2	–	Aberrant (3) NA (10)
	M2	23 (8/15)	51 (19–64)	44.8 (0.5–146.4)	5	3	4	Normal (4) Aberrant (10) NA (41)
	M3	3 (1/2)	60 (54–67)	24.8 (7.2–49.1)	1	1	–	Aberrant (2) NA (1)
	M4	13 (4/9)	52 (38–75)	51.8 (1.9–165.2)	3	2	–	Aberrant (2) NA (11)
	M5	4 (3/1)	40 (18–57)	85.6 (35.1–116)	1	1	–	Aberrant (1) NA (3)
ALL	ALL-B	8 (3/5)	42.5 (18–57)	17.9 (2.1–68.4)	–	–	–	Aberrant (3) NA (5)
AML/ALL		1 (1/0)	55	16.4	–	–	–	Aberrant (1)
All patients		66 (27/39)	50 (18–75)	36.4 (0.5–310.9)	15 (10 exclusive)	9 (4 exclusive)	6 (4 exclusive)	Normal (4) Aberrant (22) NA (40)
RNA-seq-based analysis of *NPM1* transcripts								
AML-T0	M1	9 (6/3)	52 (25–65)	65.1 (14.6–233)	4	1	–	Normal (1) Aberrant (3) NA (5)
	M2	18 (7/11)	51.5 (19–64)	37.5 (1.34–146.4)	3	3	3	Aberrant (10) NA (8)
AML-T1	M2	1 (0/1)	64	21.7	–	–	–	NA (1)
ddPCR-based analysis of *NPM1* transcripts at the three time points								
AML-T0	M1 (3), M2 (6)	9 (3/6)	52 (19–64)	59.9 (11–129.2)	2	2	2	Aberrant (5) NA (4)
AML-T1	M1 (1), M2 (8)	9 (3/6)	56 (20–64)	17.9 (2.3–35.4)	–	–	–	NA (9)
AML-T2	M1 (3), M2 (4)	7 (4/3)	54 (20–65)	15.9 (6.6–29.9)	2	–	1	NA (7)

*FLT3* mut. *FLT3*-ITD (internal tandem duplication in the *FLT3* gene), *RUNX1/RUNX1T* fusion gene, result of t(8;21) translocation, *NA* data not available, *AML-T0* AML at the time of first diagnosis, *AML-T1* AML after therapy, *AML-T2* AML at the relapse

### RNA isolation and reverse transcription

Total RNA was extracted from the PBMCs and BMMCs with a mirVana miRNA Isolation Kit (Ambion/Thermo Fisher Scientific) and DNase-treated (TURBO DNA-free kit, Ambion/Thermo Fisher Scientific). RNA integrity was evaluated using a Bioanalyzer 2100 and a Total RNA Nano Assay (Agilent Technologies, Santa Clara, CA, USA). Only RNAs with RIN (RNA Integrity Number) ≥ 7 were used for the analysis. DNA-free RNA (up to 2.5 μg per sample) was reverse transcribed using SuperScript III RT and oligo(dT) (Invitrogen, Carlsbad, CA, USA). The reaction mixtures (20 μl vol) were incubated for 1.5 h at 50 °C. After reverse transcription, the samples were incubated for 20 min at 70 °C with 10 μl of 1 M NaOH. Then, 10 μl of 1 M HCl was added for neutralization, and the cDNA was precipitated overnight at − 20 °C with 100 μl (2.5 vol) of 96% ethanol and 4 μl (1/10 vol.) of 3 M sodium acetate, pH 5.2. The centrifuged pellet was washed twice with 70% ethanol and dissolved in 50 μl of DEPC-H_2_O.

### Primer design

Due to the ddPCR product length limitation, significant sequence overlap between *NPM1* transcripts and high sequence homology with another human gene, *CLEC2D*, we were not able to design a pair of primers unique for each *NPM1* transcript. In the end, we designed three pairs of primers: one specific for *NPM1.2*, a second specific for *NPM1.3* and a third recognizing two transcripts, *NPM1.1* and *NPM1.2* (Additional file 1: Table S4, Fig. 1). One of the *NPM1.2*-specific primers crossed the exon boundary. After data collection, we subtracted the quantity of the *NPM1.2*-specific product from the result obtained in a reaction with primer pairs common for *NPM1.1* and *NPM1.2*. As a reference gene, we used *PGK1* (NM_000291.3, Gene ID 5230), coding for *Homo sapiens* phosphoglycerate kinase 1, selected as one of the most stable genes in our earlier qPCR-based analyses [30].

### Droplet digital PCR

Quantitative PCR was performed using a QX200 Droplet Digital PCR system and QX200 EvaGreen ddPCR Supermix (Bio-Rad, Hercules, CA, USA). The reaction volume was 20 μl, with a primer concentration of 250 nM, and the cDNA concentration was optimized for each sample. The PCR conditions were as follows: initial denaturation (95 °C, 5 min), 40 cycles of denaturation (95 °C, 30 s), annealing (58 °C, 30 s) and elongation (72 °C, 45 s), cooling (4 °C, 5 min), final denaturation (90 °C, 5 min) and final hold (12 °C). The temperature ramping rate was 2 °C/s. The data were analyzed with a QX200 Droplet Reader and processed in Quanta Soft v. 1.5.38.1118 (Bio-Rad). The level of each *NPM1* transcript was calculated by the Quanta Soft program as a ratio of *NPM1* droplet number to the number of *PGK1* droplets counted in a single reaction. For each sample, at least two replicate experiments were performed for each *NPM1* transcript. The ratio values from replicate experiments were averaged.

### Statistical analysis

All statistical analyses and plots were made in R ver. 3.4.1 and R Studio ver. 1.0.153. The following R packages were used: base, ggplot2, ggcorrplot, plyr, reshape2, ggsignif, ggpubr, Hmisc, and survival. Welch two sample t-tests (unpaired or paired, dependently on the data) were applied for pairwise comparisons. To test the correlations between the expression values of two genes or between a particular gene’s expression and clinical data (WBC, age and sex), Pearson’s correlations were calculated. To estimate patient outcome and correlate it with *NPM1* transcript levels, Kaplan–Meier analysis was applied. The differences between survival curves were tested with a log-rank test. The threshold p value was always set as 0.05.

### RNA-seq data analysis

RNA-seq was performed with a Genome Analyzer IIx (Illumina, San Diego, CA, USA). Up to 4 µg of total RNA extracted from PBMCs or BMMCs was used to prepare sequencing libraries with the TruSeq RNA Sample Prep Kit (Illumina). Ten pM-indexed libraries were sequenced on a single-read flow cell (TruSeq SR Cluster Kit v2 cBot, Illumina), two libraries per lane, with 72-nt long reads. The data were processed by RSEM ver. 1.3.0 for transcript quantification at the gene and gene isoform level. RSEM automatically ran STAR aligner (ver. 2.5.3a) for mapping reads to a reference genome (Homo_sapiens.GRCh38.87). For the majority of samples, only 1–2% of reads could not be aligned to the reference genome. The levels of each isoform expression were normalized first for gene length, then for sequencing depth to obtain TPM values.

## Results

### Analysis of *NPM1* transcript levels in acute leukemia at the time of diagnosis

**Fig. 2:**
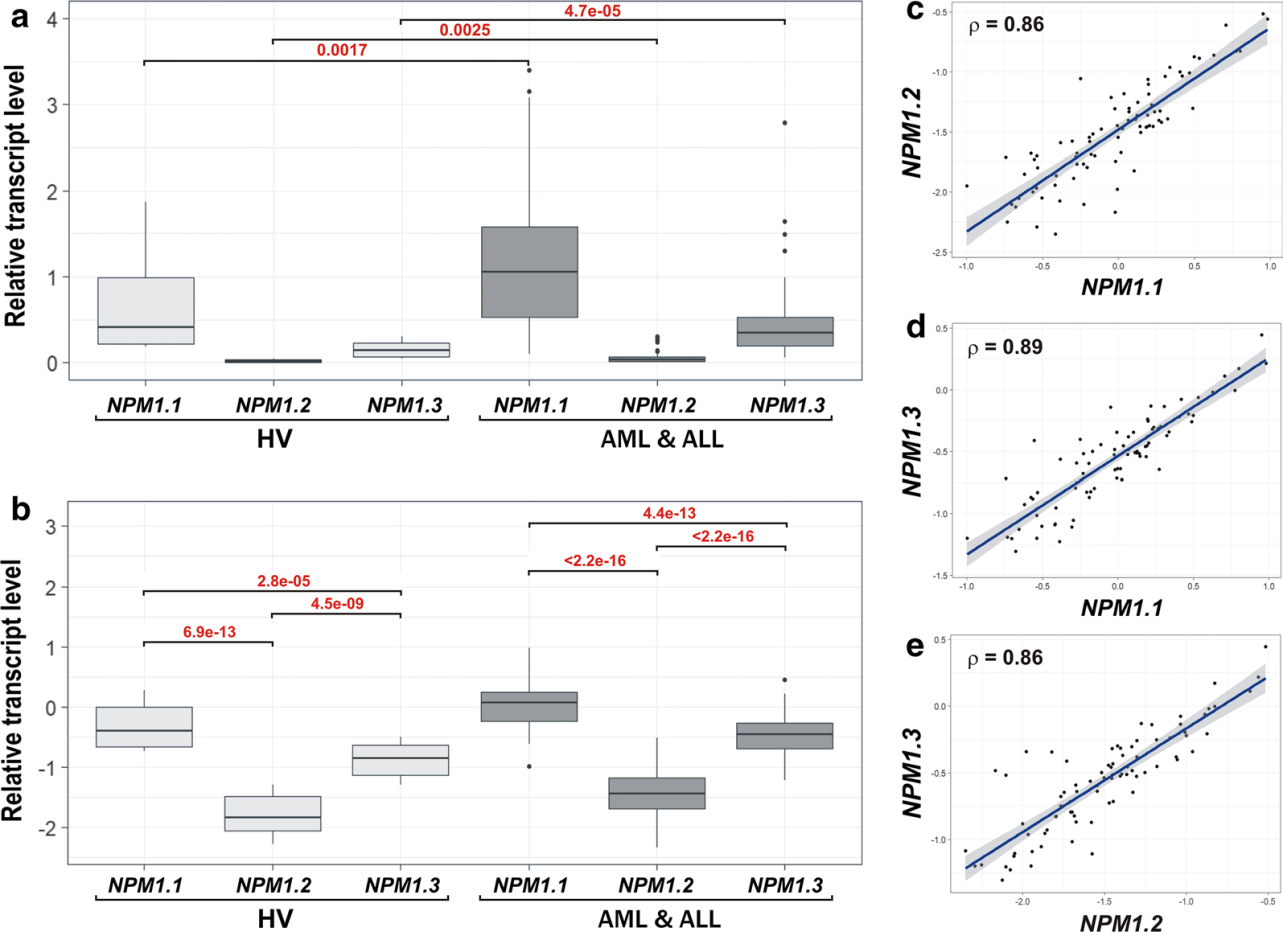
The comparison of three *NPM1* transcript levels in leukemia and HV. The results of ddPCR data analysis performed on the set of 66 acute leukemia samples and 16 HV samples, prior to (**a**) and after the logarithmic data transformation (**b**–**e**). To compare transcript levels before and after data transformation, a Wilcoxon test and unpaired t-test were used, respectively. Included p values indicate statistically significant differences. Correlation plots (**c**, **d**) include Pearson correlation coefficients

To measure the levels of *NPM1* transcripts in AML and ALL patients, we used the droplet digital PCR (ddPCR) method, allows for the absolute quantification of nucleic acid molecules in an analyzed sample [35]. To minimize the technical bias, each *NPM1* transcript was measured together with a reference gene (*PGK1)* in a single reaction (Additional file 1: Figure S1). In total, we analyzed the levels of three *NPM1* transcripts, *NPM1.1*, *NPM1.2* and *NPM1.3*, in 66 mononuclear cell samples (extracted from peripheral blood, PB, or bone marrow, BM) collected at the time of the first diagnosis from adult patients with acute leukemia and from 16 adult healthy volunteers (HV). Patient samples included 57 AML, 8 ALL and one biphenotypic leukemia (Table 1, Additional file 1: Table S2). Analysis of ddPCR results revealed the evident differences in the levels of the particular *NPM1* transcripts in all samples, either leukemia or HV (Fig. 2a, b, Table 2). All *NPM1* transcripts were increased in leukemia compared to HV. The proportions between levels of particular transcripts were similar in leukemia and HV. The most abundant transcript was *NPM1.1*, whose median level was approximately 30 times higher than the median level of *NPM1.2* and approximately three times higher than the median level of *NPM1.3*. Because the transcript levels did not follow Gaussian distribution, we subjected the data to log_10_-transformation prior to further statistical analysis. Nevertheless, the differences between the levels of particular transcripts and the differences between leukemia and HV were statistically significant before and after data transformation, which was supported by the appropriate tests. As shown in Fig. 2c–e, the levels of the studied *NPM1* transcripts were also highly correlated, which was reflected by high Pearson correlation coefficients (0.89 for *NPM1.1* and *NPM1.3*; 0.86 for *NPM1.1* and *NPM1.2;* 0.86 *NPM1.2* and *NPM1.3*).

**Table 2:** Median expression of three protein-coding NPM1 transcripts in leukemia and HV, and relationships between particular transcript levels

	*NPM1.1* median level	*NPM1.2* median level	*NPM1.3* median level	*NPM1.1*/*NPM1.2* ratio	*NPM1.1*/*NPM1.3* ratio	*NPM1.3*/*NPM1.2* ratio
Leukemia	1.171	0.035	0.350	33.5	3.3	10.0
HV	0.410	0.014	0.140	29.3	2.9	10.0
Leukemia/HV ratio	2.9	2.5	2.5	1.1	1.1	1.0

**Fig. 3:**
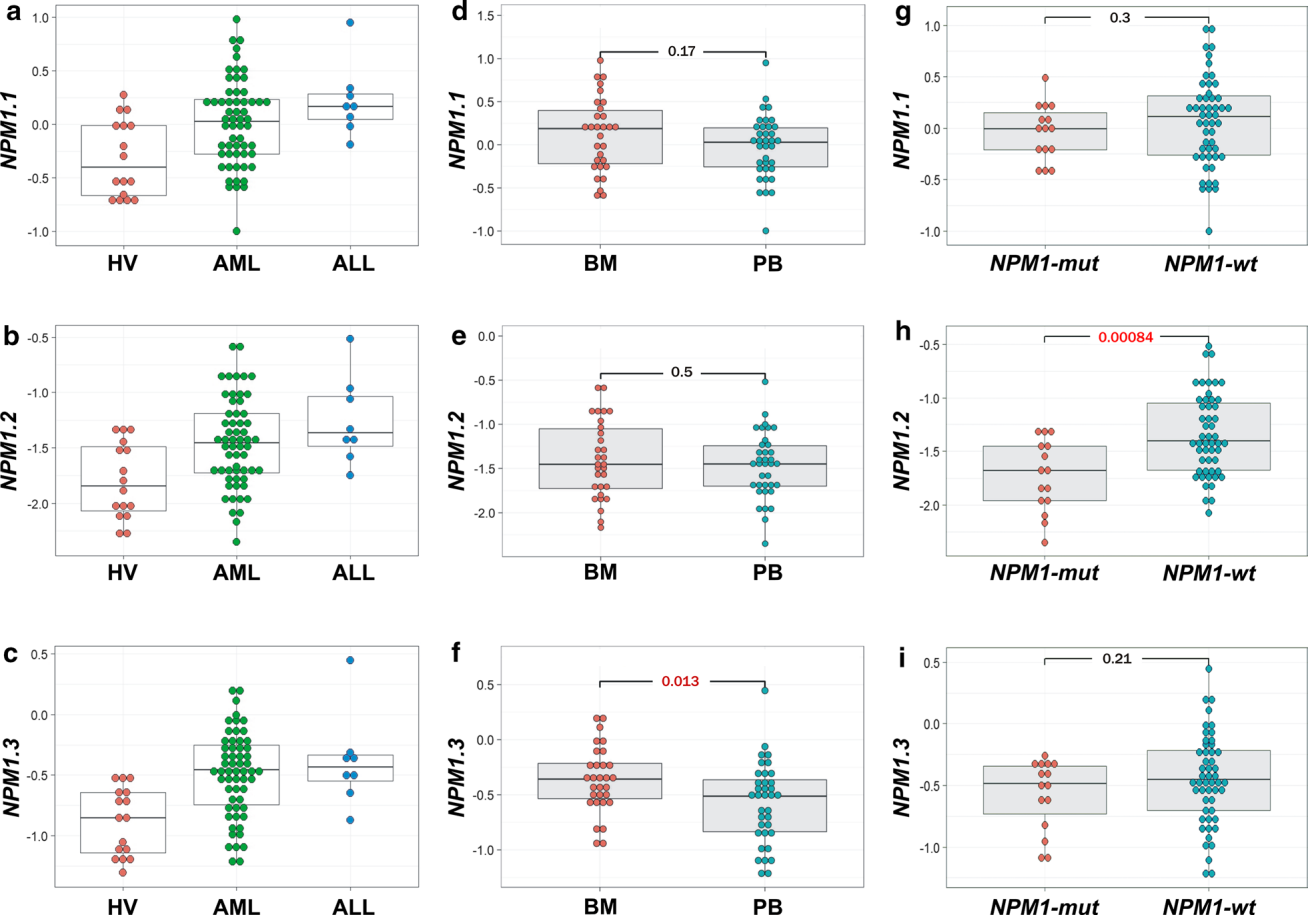
The comparison of three *NPM1* transcript levels in leukemia samples stratified by the disease types (**a**–**c**), source tissue type (BM or PB) (**d**–**f**), and *NPM1* mutation status (**g**–**i**). Each dot represents one sample. Background boxplots show the median (a line in the middle) and the first and third quartiles (the bottom and top of the box). Due to the small number of ALL samples, statistical analysis was not applied to the plots **a**–**c**. Plots **d**–**i**, drawn excluding HV samples, include t-test p values (statistically significant indicated in red)

After dividing the leukemia samples into AML and ALL, we noted similar levels of all *NPM1* transcripts in both types of leukemia. Consequently, differences were observed between each disease and HV (Fig. 3a–c). Similarly, we did not observe significant differences in *NPM1* transcript levels between AML FAB subtypes (Additional file 1: Figure S2A–C). Because the studied cell samples were extracted from PB or BM, we tested whether *NPM1* transcript levels depended on tissue type. Comparing the patient samples, we found a significantly higher level of *NPM1.3* transcript in BM versus PB (Fig. 3f). The levels of *NPM1.1* and *NPM1.2* did not differ between PB and BM (Fig. 3d, e).

To test whether *NPM1* expression depends on the mutation status, we divided the studied leukemia samples into the following groups: (i) *NPM1*-mut leukemia (with a tetranucleotide insertion in exon 12 of the *NPM1* gene, 15 samples) vs. leukemia without an *NPM1* mutation (wt, 51 samples); (ii) *FLT3*-mut leukemia (with *FLT3*-ITD, 9 samples) vs. leukemia without a *FLT3* mutation (wt, 57 samples); and (iii) leukemia with a *RUNX1/RUNX1T1* fusion gene, generated as a result of translocation t(8;21) (4 samples) vs. leukemia samples without this translocation (wt, 62 samples). As shown in Fig. 3h, we observed a statistically significant decrease of *NPM1.2* transcript in *NPM1*-mut samples compared to the samples without an *NPM1* mutation. The level of *NPM1.2* transcript in *NPM1*-mutated leukemia was close to the level noted for HV. The levels of *NPM1.1* and *NPM1.3* did not differ between leukemia samples divided according to *NPM1* mutation status (Fig. 3g, i). We did not observe a significant impact of the *FLT3* mutation on the level of *NPM1* transcripts, although the level of *NPM1.2* was slightly lower in *FLT3*-mutated samples vs. samples without this mutation (Additional file 1: Figure S2D–F). In the samples with *RUNX1/RUNX1T1*, the levels of *NPM1* transcripts were higher compared to the samples without t(8;21), but the number of samples with *RUNX1/RUNX1T1* was too small to perform statistical analysis (Additional file 1: Figure S2G–I). As all samples with t(8;21) were extracted from BM cells, the plots were drawn excluding PB samples.

Analyzing the impact of the white blood cell (WBC) count, sex and age of patients, we found no influence of these variables on the level of *NPM1* transcripts (Additional file 1: Table S5).

### Impact of *NPM1* transcript levels on patient outcome

**Fig. 4:**
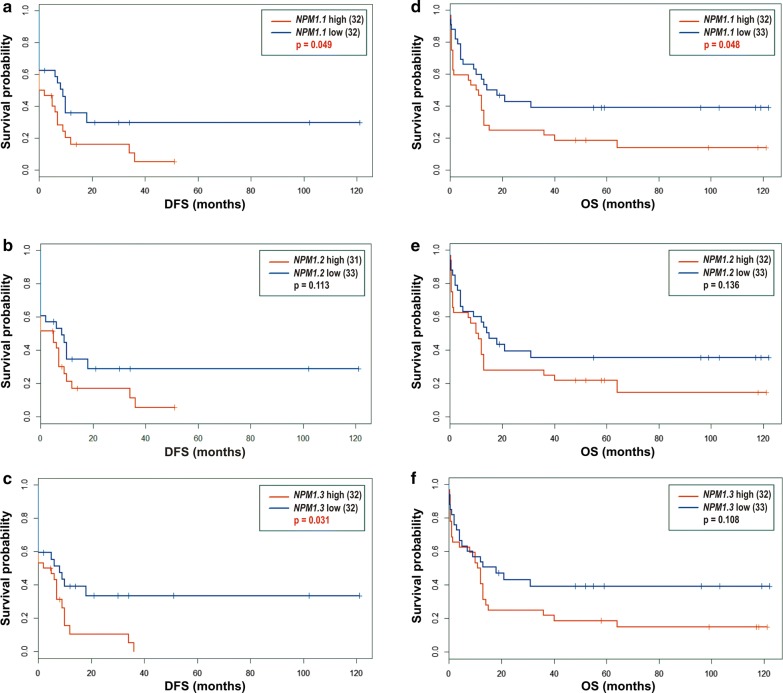
Disease free survival (DFS) (**a**–**c**) and overall survival (OS) (**d**–**f**) of 66 leukemia patients divided according to the level of *NPM1.1* (**a**, **d**), *NPM1.2* (**b**, **e**) and *NPM1.3* (**c**, **f**) transcripts. Log-rank test p values < 0.05 indicate statistically significant differences between two curves

As the patients were recruited starting from 2007, we were able to monitor their outcome for more than 10 years. Forty-seven out of 66 patients died during this time, and the remaining patients are still alive or stopped contacting the clinic. To evaluate the impact of *NPM1* transcript levels measured at the time of diagnosis and other variables (mutation status, WBC, sex) on further patient outcome, we applied Kaplan–Meier analysis. In the entire group of patients, median disease-free survival (DFS) and median overall survival (OS) were equal to 2 and 12 months, respectively (Additional file 1: Figure S3A, B). To analyze the impact of *NPM1* transcript levels on DFS and OS, we dichotomized samples based on the median level of the particular *NPM1* transcript. The results are shown in Fig. 4 and Additional file 1: Table S6. For all three *NPM1* transcripts, a high level of expression was associated with shorter DFS and shorter OS. However, the differences between the two survival curves were statistically significant only for *NPM1.1* and *NPM1.3* levels in the case of DFS (Fig. 4a, c) and for *NPM1.1* in the case of OS (Fig. 4d). *NPM1* and *FLT3* mutation status and sex had no impact on DFS and OS (Additional file 1: Figure S3C–F, I, J, Table S6). From the clinical features, only the WBC count seemed to be relevant for patient outcome. Patients with high WBC number had shorter DFS and OS, although the difference between DFS curves was less evident (p = 0.054) (Additional file 1: Figure S3 G, H, Table S6).

### RNA-seq-based analysis of the expression of the *NPM1* gene and corresponding transcripts

To verify the results described above and extend our research to additional *NPM1* transcripts, we took advantage of next generation sequencing of transcriptomes (RNA-seq). This approach enabled us to examine the expression of the *NPM1* gene and corresponding transcripts on the background of other genes and transcripts present in leukemic cells. The data set included 27 samples collected at the time of diagnosis (T0 time point) from AML patients with M1 (9 patients) and M2 (18 patients) FAB types, referred to as AML-T0, and one sample collected from an AML-M2 patient 2 months after therapy (T1 time point), referred to AML-T1 (Table 1, Additional file 1: Table S2). Control samples were represented by one BM HV sample and one pool of 12 PB HV samples. Ranking genes according to the normalized expression values (TPM, transcripts per million) demonstrated that the *NPM1* gene is one of the most abundant genes transcribed in BM and PB mononuclear cells, with median ranking position 124 in AML-T0, 188 in AML-T1 and 181.5 in HV per approximately 14,000 genes detected with TPM > 1. The TPM of the *NPM1* gene was equal to 1492 in AML-T0 (median), 752 in AML-T1 and 799 in HV (mean), indicating a twofold increase in leukemia at the time of diagnosis and a decrease after therapy to the level typical for HV.

**Fig. 5:**
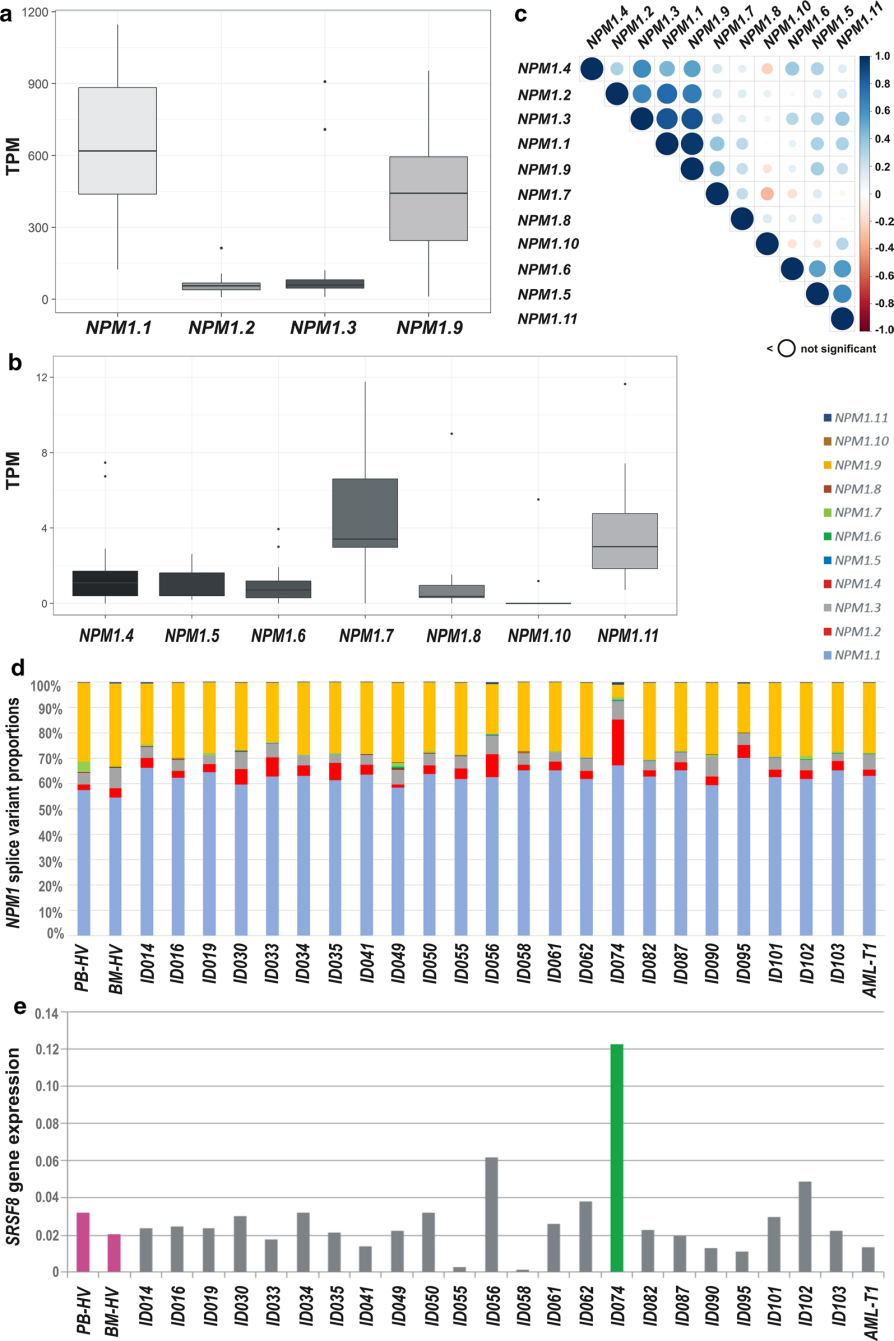
The comparison of *NPM1* transcript levels estimated from the analysis of RNA-seq data. Boxplots A and B present TPM values. For clear presentation, *NPM1* transcripts were divided into two plots according to the level of expression: (**a**) high and (**b**) low. Boxplots do not show 8 outliers for *NPM1.1*, 6 outliers for *NPM1.7*, and 2 outliers for *NPM1.10*. A correlation plot (**c**) shows Spearman correlation coefficients for each pair of *NPM1* transcripts, ranked according to the similarity of expression. Color intensity and the size of the circles are proportional to the correlation coefficients. Circles smaller than an empty one under the plot means not statistically significant correlation. **d** The ratio of *NPM1* splice variants in particular samples. Legend is above the plot. **e** Expression level of *SRSF8* gene, encoding one of the splicing factors from the Ser/Arg-rich protein family. Control samples are indicated in pink (BM-HV—bone marrow sample from a healthy volunteer; PB-HV—a pool of 12 peripheral blood samples from healthy volunteers) whereas AML samples in grey (ID014-ID103—AML at the time of first diagnosis, AML-T1—AML sample after therapy), except for one sample (ID074, green) with extremely higher level of *SRFS8* expression

Performing analysis on a transcript (gene isoform) level, we were able to detect all 11 *NPM1* transcripts presented in Fig. 1, though one transcript (*NPM1.10*) was present in only four samples, which resulted in a median level of 0 (Table 3, Fig. 5a, b). We observed high differences between the levels of *NPM1* transcripts but the contribution of particular transcripts to the total *NPM1* gene expression was generally similar in all samples (Fig. 5d). Consistent with the ddPCR results, the most abundant *NPM1* transcript was *NPM1.1* (Fig. 5a). The median position of this transcript was equal to 160 in AML-T0, 254 in AML-T1 and 277 in HV per approximately 38,000 splice variants detected with TPM > 1. *NPM1.1* was responsible for 63% of the total *NPM1* gene expression in AML samples and 56% in HV samples. *NPM1.2* and *NPM1.3* together constituted 8–9% of the total *NPM1* gene expression in all samples. RNA-seq data revealed a significant contribution of an additional transcript, *NPM1.9* (approximately 30% in both AML and HV samples) (Fig. 5a). The remaining *NPM1* transcripts were transcribed at low to marginal levels (Fig. 5b, d). The levels of the four most abundant *NPM1* transcripts and *NPM1.4* were highly correlated with each other (Fig. 5c).

**Table 3:** RNA-seq-based comparison of *NPM1* transcript levels in AML and HV samples

Transcript	AML-T0		AML-T1		HV		AML-T0/HV TPM ratio
	Median level (TPM)	Contribution to the total *NPM1* gene expression (%)	Median level (TPM)	Contribution to the total *NPM1* gene expression (%)	Median level (TPM)	Contribution to the total *NPM1* gene expression (%)	
*NPM1.1*	950.37	63.68	473.63	63.02	445.485	55.73	2.13
*NPM1.2*	55.36	3.71	18.14	2.41	24.655	3.084	2.25
*NPM1.3*	62.88	4.21	45.8	6.09	52.505	6.569	1.20
*NPM1.4*	1.09	0.073	0.43	0.057	1.46	0.183	0.75
*NPM1.5*	0.76	0.051	0.37	0.049	0.655	0.082	1.16
*NPM1.6*	0.71	0.048	0.5	0.067	0.53	0.066	1.34
*NPM1.7*	4.53	0.304	3.07	0.408	13.785	1.725	0.33
*NPM1.8*	0.4	0.027	0	0	0.385	0.048	1.04
*NPM1.9*	412.59	27.65	206.85	27.52	256.25	32.06	1.61
*NPM1.10*	0	0	0	0	0	0	–
*NPM1.11*	3.7	0.248	2.77	0.369	3.61	0.452	1.02
total	1492.39	100	751.56	100	799.32	100	–

Comparing particular AML samples, we noticed some fluctuations in the level of *NPM1.2* and *NPM1.9* (Fig. 5d). The extreme case was AML sample ID074, which presented the most outstanding proportion of these two splice variants (the highest level of *NPM1.2* and the lowest level of *NPM1.9* comparing to all other samples). To test whether the aberrant proportions of *NPM1* splice variants could be linked to the expression level of splicing factor coding genes, we retrieved from our RNA-seq data the results of expression of *SRSF* (Ser-Arg-rich Splicing Factor) gene family members. The rationale of this analysis was overexpression of *SRSF1* gene, encoding an alternative splicing regulator, in different cancer types, described as one of the features promoting cancerogenesis [36]. Comparing the levels of twelve *SRSF* genes in the studied samples, we found the expression of six genes (*SRSF2*, *SRSF3*, *SRSF4*, *SRSF10*, *SRSF11*, and *SRSF12*) was rather decreased in AML when compared to the control BM. For the remaining *SRSF* genes, we did not observe substantial differences between HV and AML samples, except one AML sample (ID074 mentioned above) which revealed higher levels of *SRSF1*, *SRSF2*, *SRSF5*, *SRSF7*, *SRSF8*, and *SRSF9*. The most evident upregulation was observed for the *SRSF8* gene (Fig. 5e). Another sample with increased *SRSF8* level (ID056), presented similar aberration in *NPM1.2* and *NPM1.9* proportion as the ID074 sample, however, the effect was not so spectacular. It can suggest the association between *NPM1* splicing dysfunction and deregulated expression of splicing-related genes.

As shown in Table 3 and Additional file 1: Figure S4, RNA-seq analysis confirmed an increase of all three protein-coding *NPM1* transcripts in AML-T0 compared to HV. Moreover, we found that *NPM1.9*, the most abundant non-coding transcript, was also increased in AML (Additional file 1: Figure S4D). Interestingly, in AML-T1, the levels of all detected *NPM1* transcripts were similar or even lower than those observed in HV, suggesting that the level of *NPM1* transcripts increases at the time of first AML diagnosis and decreases after therapy. Similarly as in the ddPCR data analysis, we noted a higher level of *NPM1* transcripts in BM than PB (Additional file 1: Figure S4E–H) and in AML samples with t(8;21) compared to the samples without this translocation (Additional file 1: Figure S4I–L).

The discrepancies between ddPCR and RNA-seq analyses were somewhat different proportions between the levels of *NPM1.1*, *NPM1.2* and *NPM1.*3 transcripts. This discrepancy most likely results from mapping short RNA-seq reads that cannot unequivocally distinguish some of the transcripts (the same exon-exon boundaries occur in different transcripts). On average, only 20% of reads were unequivocally mapped whereas 78% of reads mapped to multiple transcripts and 2% were unalignable.

### Analysis of *NPM1* transcripts in AML samples collected after therapy and at relapse

**Fig. 6:**
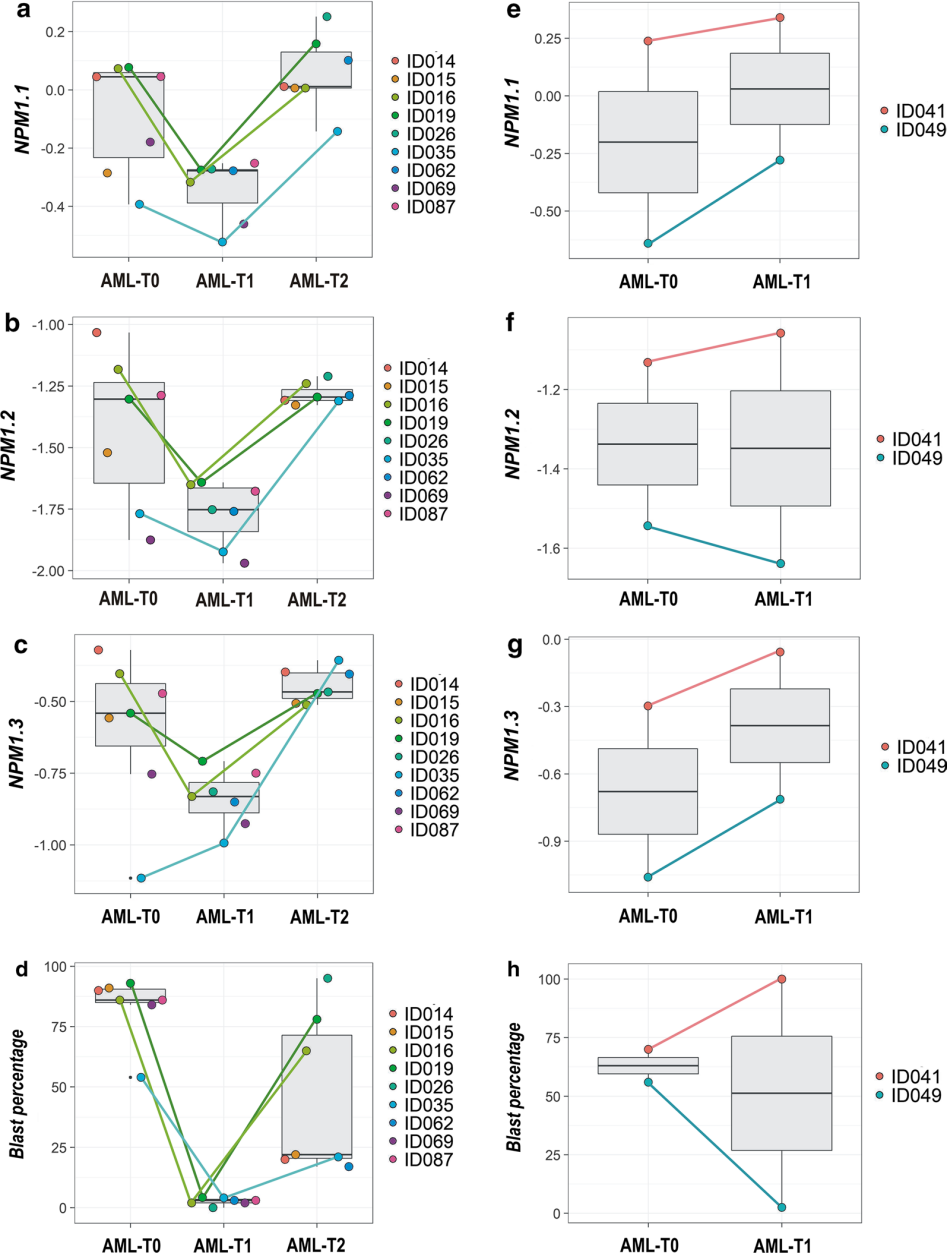
The comparison of three *NPM1* transcript levels in AML samples collected at three time points: T0 (at the time of first diagnosis), T1 (after treatment), and T2 (at relapse). Plots **a**–**c** present samples from patients who reached complete remission after treatment. Samples collected from the same patients at all three time points are indicated with dot-connecting lines. Plots **e**–**g** present samples from patients who were therapy-resistant. Plots **d** and **h** present the percentage of blasts in the corresponding sample sets. Each dot represents one sample. Background boxplots show the median (a line in the middle) and the first and third quartiles (the bottom and top of the box)

Because the levels of three protein-coding *NPM1* transcripts were increased in leukemia compared to HV and RNA-seq data analysis suggested the decrease of *NPM1* transcript level after therapy, we used ddPCR to test how the levels of three protein-coding *NPM1* transcripts changed at three time points: at the time of first diagnosis (AML-T0), after therapy (AML-T1) and at relapse (AML-T2). The study included 11 patients, but samples from all three time points were available only for three patients. In total, we were able to compare 9 AML-T0, 9 AML-T1 and 7 AML-T2 samples (Table 1, Additional file 1: Table S3). The obtained results showed the decrease of all three *NPM1* transcripts after therapy and an increase at relapse, to the level comparable to that observed at the time of diagnosis. A paired t-test applied for the samples collected at two time points from the same patients, showed the difference in the transcript level was statistically significant between AML-T1 and AML-T2 for all three *NPM1* transcripts and between AML-T0 and AML-T2 for *NPM1.1* and *NPM1.2*. (Additional file 1: Figure S5). *NPM1* suppression after therapy was observed for all patients who reached complete remission (Fig. 6a–c). Contrary, in the case of two patients resistant to therapy, the level of *NPM1* transcripts even increased in T1 time point when compared to T0 (Fig. 6e–g). The changes in *NPM1* expression can be correlated with the blast percentage (and to a lesser extent with WBC count) of the samples collected at the first diagnosis, after therapy and at relapse (Fig. 6d, i). For the patients who reached complete remission, Pearson correlation coefficients for the blast percentage and *NPM1.1*, *NPM1.2* and *NPM1.3* levels measured at the three time points were equal to 0.6, 0.56 and 0.47, respectively. For the therapy-resistant patients, Pearson correlation coefficients for the blast percentage and *NPM1.1*, *NPM1.2* and *NPM1.3* levels measured at the two time points were equal to 0.6, 0.88 and 0.62, respectively.

## Discussion

Regarding AML, only one study dedicated to *NPM1* splice variants has been reported to date [31]. Although the authors showed an increase of three *NPM1* transcripts in AML compared to HV, they focused mainly on the *NPM1.3* transcript (R2 in their study). Our results confirmed the increase of all three *NPM1* transcripts in acute leukemia. In the case of two transcripts, *NPM1.1* and *NPM1.3*, the level of upregulation in AML vs. HV was practically the same (ratio 2.9 for *NPM1.1* and 2.5 for *NPM1.3* from our study versus ratios 3.15 and 2.97, respectively, from the study by Zajac et al. [31]). However, the level of *NPM1.2* transcript and *NPM1.2* ratio between AML and HV was much higher in the work of Zajac et al. [31]. Consequently, the proportions between particular transcripts were different than observed in our study. We show, using the highly sensitive ddPCR method, that *NPM1.2* is transcribed at the lowest level compared to *NPM1.1* and *NPM1.3* and that the *NPM1.2* level in HV is barely detectable.

According to Grisendi et al. [37], Ruggero et al. [38] and Falini et al. [1], aberrantly increased *NPM1* can act as an oncogene, promoting cell growth through enhanced ribosome production and cell survival through cell death inhibition. Described here, increased levels of the protein-coding *NPM1* transcripts in leukemia support the association of *NPM1* gene expression with leukemogenesis. The lack of difference in *NPM1* transcript levels between AML and ALL suggests a more general mechanism, not limited to the myeloid lineage. The role of *NPM1* in oncogenesis may be additionally supported by the observed here decrease of the *NPM1* transcripts after therapy and an increase at relapse. The decrease of the *NPM1* levels upon treatment was also demonstrated in liver cancer [39], breast cancer [40] and lung cancer cells [25]. Our results suggest the decrease of *NPM1* expression in a complete remission is a consequence of the blast proliferation suppression by the therapeutic agents. It is not surprising that in the therapy-resistant samples, where the number of leukemic blasts is still high, high *NPM1* transcript levels are maintained.

The fact that the levels of particular *NPM1* transcripts were highly correlated with each other indicates common transcriptional regulation. However, we also found some differences in the expression pattern of *NPM1* transcripts. Of note, the elevated level of only one transcript, *NPM1.2*, was associated with the absence of an *NPM1* mutation. The *NPM1.3* level was higher in BM than in PB and seemed to be upregulated in samples with t(8;21). Moreover, RNAseq data analysis revealed the existence of variation in the proportions of particular *NPM1* splice variants between the studied samples. This can be influenced by the mutations in genes encoding splicing factors. As the co-occurrence of different mutations is often found in AML, we cannot exclude some of our patients carry spliceosome mutations. Also, mutations in canonical splice sites (exon/intron boundaries) and in splicing regulatory sequences of *NPM1* may affect proportions between the alternative transcripts. To test it, exome sequencing would be necessary. However, not only mutations but also changes of splicing-related gene expression can impact the levels of alternative splice variants. For example, overexpression of alternative splicing regulator, *SRSF1*, detected in different cancer types, was postulated to promote cancerogenesis [36]. Interestingly, in AML, decreased expression of *SRSF* family members was shown [41] what is generally consistent with our observations. From the other side, *SRSF1* and few other *SRSF* genes, were clearly increased in the AML sample with the highest level of *NPM1.2* and the lowest level of *NPM1.9* comparing to all other samples, AML and HV. The functions of these two *NPM1* transcripts are unknown, but *NPM1.2* encodes a protein similar to the most abundant NPM1 protein, with the same C-end, including region required for nucleolar localization and nucleocytoplasmic shuttling of the protein. Therefore, the functions of *NPM1.1* and *NPM1.2* could be similar. Contrary, *NPM1.9* is a short non-coding transcript, containing only two first exons and 5′ part of the following intron. Translation of this transcript would generate a short fragment of the protein N-end, linked to the oligomerization domain, responsible also for the interactions with other proteins. Other functional domains, implicated in nucleic acid and histone binding, ATP binding and ribonuclease activity, are localized in the central and C-end regions of NPM1 protein [1, 2, 33, 42]. The high level of *NPM1.9,* detected in both, AML and HV samples, suggests this transcript may play an important (e.g. regulatory) role at the RNA level.

Survival analysis of our patients showed that the expression level of *NPM1* transcripts affected patient outcome more than the presence or absence of *NPM1* mutation documented earlier [11, 43]. The differences between survival curves drawn for patients with high and low levels of *NPM1.1* and *NPM1.3* transcripts were comparable to the difference between survival curves drawn for patients stratified according to the WBC count, the only clinical parameter influencing the outcome of patients from our study. Importantly, our data revealed that a high level of *NPM1* transcripts correlates with worse prognosis. This finding is consistent with the report by Leotoing et al. [44], who showed that a high level of *NPM1* enhances the aggressiveness of prostate tumors. Recently, the association of the high *NPM1* expression with poor prognosis was also demonstrated in bladder urothelial carcinoma [45], gastric cancer [46] and glioma [47]. Surprisingly, in the study by Zajac et al. [31], the effect was opposite: low expression was associated with worse prognosis. However, this observation was limited to *NPM1.3* and NK-AML patients. Therefore, the observed discrepancies between the study by Zajac et al. [31] and other studies, including ours, may be a consequence of karyotype-based patient selection. Zajac et al. [31] demonstrated a relationship between *NPM1.3* transcript expression with *NPM1* mutation and patient outcome only in a subgroup of NK-AML patients, constituting half of the entire studied cohort. Our study included 1/3 of samples with cytogenetic abnormalities, only a few samples with normal karyotype, and for more than 60% of patients, the karyotype was unknown. The low number of *NPM1*-mutated samples (23%) in our study is typical for unselected primary AML patients, for which no association between *NPM1* mutation and prognosis exists. Additionally, the relation between *NPM1* mutation and prognosis may be even more complex. Some authors reported no difference in the rate of complete remission between NK-AML patients with and without *NPM1* mutation [48], and others noted that the impact of *NPM1* mutation on prognosis could be dependent on the age of patients [10] or the presence of additional mutations, e.g., in the *IDT1* gene [49]. These findings demonstrate that the influence of *NPM1* mutation and expression on patient outcome should be further studied.

Our ddPCR-based analysis of three *NPM1* protein-coding transcripts was supplemented with the analysis of RNA-seq data and the results were generally consistent. NGS-based transcriptome studies, which are currently extensive, can be an invaluable source of knowledge on alternative gene variants. The advantage of RNA-seq is localization of a gene/transcript of interest on a background of all genes transcribed in a cell. We demonstrated that the *NPM1* gene and *NPM1.1* transcript were among the most abundant genes and transcripts. However, due to NGS technology limitations, the results of the RNA-seq data analysis should be interpreted with caution. *NPM1* is among the genes with a high number of alternative transcripts. Because many exons are shared between *NPM1* transcripts, some transcripts cannot be reliably distinguished and quantified.

## Conclusions

We showed that the *NPM1* gene and its predominant transcripts are increased in acute leukemia when compared to healthy control samples. Furthermore, we showed that the levels of particular *NPM1* transcripts are highly correlated with each other, and the expression levels of at least two of them can be associated with AML patient outcome. Our results suggest the low level of *NPM1* expression predicts better prognosis. In our studied group, including AML with different karyotypes, the level of *NPM1* expression affected patient outcome more than *NPM1* mutation. RNA-seq data analysis revealed the spectrum of *NPM1* splice variants in cells is wider than it would appear from previous studies. Aberrant proportions of particular *NPM1* transcripts could be linked to abnormal expression of genes encoding alternative splicing (AS) factors.

## Additional file

**Additional file 1.** Additional tables and figures.

## Abbreviations

NPM1: nucleophosmin 1
AML: acute myeloid leukemia
NK-AML: normal karyotype AML
ALL: acute lymphoblastic leukemia
HV: healthy volunteer
*FLT3*-ITD: FMS-like tyrosine kinase 3-internal tandem duplication
ddPCR: droplet digital polymerase chain reaction
PBMCs: peripheral blood mononuclear cells
BMMCs: bone marrow mononuclear cells
DFS: disease-free survival
OS: overall survival
WBC: white blood cell
CR: complete remission
TPM: transcripts per million
