# Supplementary material for: NPM1 alternative transcripts are upregulated in acute myeloid and lymphoblastic leukemia and their expression level affects patient outcome
Source: J Transl Med. 2018 Aug 20;16:232. doi: 10.1186/s12967-018-1608-2 (PMC6102803; doi:10.1186/s12967-018-1608-2)
Supplement: Supplementary file 1 — Additional file 1. Additional tables and figures. [file 12967_2018_1608_MOESM1_ESM.zip › Additional file 1.pdf]

Additional tables and figures

Table S1 The list of all known human *NPM1* gene splice variants, based on Ensembl and NCBI databases.

Ensemble Transcript Name	Ensemble Transcript ID (UniProt ID)	Ensemble Transcript Length (Protein Length)	NCBI Transcript Name (Protein Name)	NCBI RefSeq	NCBI Transcript Length (Protein Length)	**Transcript Name used in this study**
NPM1-201	ENST00000296930.9 (A0A0S2Z491)	1758 bp (294 aa)	variant 1 (isoform 1)	NM_002520.6 (NP_002511.1)	1449 bp (294 aa)	***NPM1.1***
NPM1-202	ENST00000351986.10 (A0A0S2Z4G7)	1237 bp (265 aa)	variant 2 (isoform 2)	NM_199185.3 (NP_954654.1)	1362 bp (265 aa)	***NPM1.2***
NPM1-203	ENST00000393820.2 (A0A140VJQ2)	1598 bp (259 aa)	variant 3 (isoform 3)	NM_001037738.2 (NP_001032827.1)	1347 bp (259 aa)	***NPM1.3***
NPM1-204	ENST00000517671.5 (A0A0S2Z491)	1338 bp (294 aa)	variant 7 (isoform 7)	NM_001355006.1 (NP_001341935.1)	1339 bp (294 aa)	***NPM1.1***
NPM1-205	ENST00000518587.1	670 bp (non-coding)				***NPM1.4***
NPM1-206	ENST00000519955.1	571 bp (non-coding)				***NPM1.5***
NPM1-207	ENST00000521260.1	510 bp (non-coding)				***NPM1.6***
NPM1-208	ENST00000521672.5 (E5RI98)	597 bp (111 aa)				***NPM1.7***
NPM1-209	ENST00000521710.1	386 bp (non-coding)				***NPM1.8***
NPM1-210	ENST00000523339.1	268 bp (non-coding)				***NPM1.9***
NPM1-211	ENST00000523622.1 (E5RGW4)	247 bp (59 aa)				***NPM1.10***
NPM1-212	ENST00000524204.1	640 bp (non-coding)				***NPM1.11***
			variant 4 (isoform 4)	NM_001355007.1 (NP_001341936.1)	1369 bp (230 aa)	
			variant 5 (isoform 5)	NM_001355009.1 (NP_001341938.1)	1260 bp (230 aa)	

			variant 6 (isoform 6)	NM_001355010.1 (NP_001341939.1)	1068 bp (167 aa)	
			variant 8	NR_149149.1	1321 bp (non-coding)	

Table S2 The list of all analyzed samples and data (separate xlsx file)

Table S3 ID of patient samples included into the time point experiment.

No.	T0	T1	T2
1	ID014-T0		ID014-T2
2	ID015-T0		ID015-T2
3	ID016-T0	ID016-T1	ID016-T2
4	ID019-T0	ID019-T1	ID019-T2
5		ID026-T1	ID026-T2
6	ID035-T0	ID035-T1	ID035-T2
7	ID041-T0	ID041-T1*	
8	ID049-T0	ID049-T1*	
9		ID062-T1	ID062-T2
10	ID069-T0	ID069-T1	
11	ID087-T0	ID087-T1	

The time point experiment included 11 patients, but samples from all three time points (T0 – first diagnosis; T1 – after therapy; T2 – relapse) were available only for three patients (indicated in red color). For the rest of patients, samples in two different time points could be compared: T0 and T2 (2 patients, blue color), T1 and T2 (2 patients, green color), and T0 and T1 (4 patients, purple color). 2 patients (indicated with stars) were resistant to therapy, therefore, complete remission was not achieved.

Table S4 Primers used in ddPCR analysis.

Transcript	ddPCR primer sequences	Product length	Ensemble transcript compatibility
NPM1.1	Forward<br>5’- GGACAAGAATCCTTCAAGAAACAG -3’<br>Reverse<br>5’- TGGACAACATTTATCAAACACGG -3’	345 bp	ENST00000296930<br>ENST00000517671<br>ENST00000351986
NPM1.2	Forward<br>5’- GCGCCAGTGAAGAAAGGACAA -3’<br>Reverse<br>5’- GACTTCCTCCACTGCCAGAGAT -3’	224 bp	ENST00000351986
NPM1.3	Forward<br>5’- GCGCATTGAACAGTCCTGGG -3’<br>Reverse	172 bp	ENST00000393820

	5’- CCAGCCTGAAGAGGCATGGGT -3’		
*PGK1**	Forward<br>5’- GGGAAAAGATGCTTCTGGGAA -3’<br>Reverse<br>5’- TTGGAAAGTGAAGCTCGGAAA -3’	75 bp	ENST00000373316.4<br>ENST00000491291.1

*reference gene, encoding phosphoglycerate kinase 1.

Table S5 The results of correlation analysis of *NPM1* transcript levels and WBC count, age and sex of patients.

Transcript	***NPM1.1***		***NPM1.2***		***NPM1.3***	
Variable	Pearson correlation coefficient	p value	Pearson correlation coefficient	p value	Pearson correlation coefficient	p value
WBC count	0.0598	0.5959	0.0837	0.4576	0.1689	0.1316
Age	0.0030	0.9792	-0.0617	0.5865	0.0240	0.8324
	ANOVA p value		ANOVA p value		ANOVA p value	
Sex (F vs M)	0.775		0.74		0.806	

Table S6 Median DFS and OS of leukemia patients divided into groups according to the level of the studied *NPM1* transcripts, *NPM1* mutation status, *FLT3* mutation status and the number of WBC.

Group of patients	Median DFS (months)	DFS - p value	Median OS (months)	OS - p value	Median transcript level
NPM1.1 high	1	**0.049**	10.5	**0.048**	1.843 (*NPM1.1*)
NPM1.1 low	9		18		0.562 (*NPM1.1*)
NPM1.2 high	5	0.113	10.5	0.136	0.066 (*NPM1.2*)
NPM1.2 low	8		15		0.020 (*NPM1.2*)
NPM1.3 high	3.5	**0.031**	11.5	0.108	0.552 (*NPM1.3*)
NPM1.3 low	8		18		0.188 (*NPM1.3*)
all patients	6		12		See Table 2
NPM1-mut	2	*	13	*	-
NPM1-wt	6		12		-
FLT3-mut	9	*	10	*	-
FLT3-wt	6		12		-
					Median WBC
WBC high	0	0.054	5	**0.025**	74
WBC low	9		18		9.7
Sex - F	5	0.457	12.5	0.97	-
Sex - M	6		12		-

*Due to the small sample size, statistical analysis was not applied.

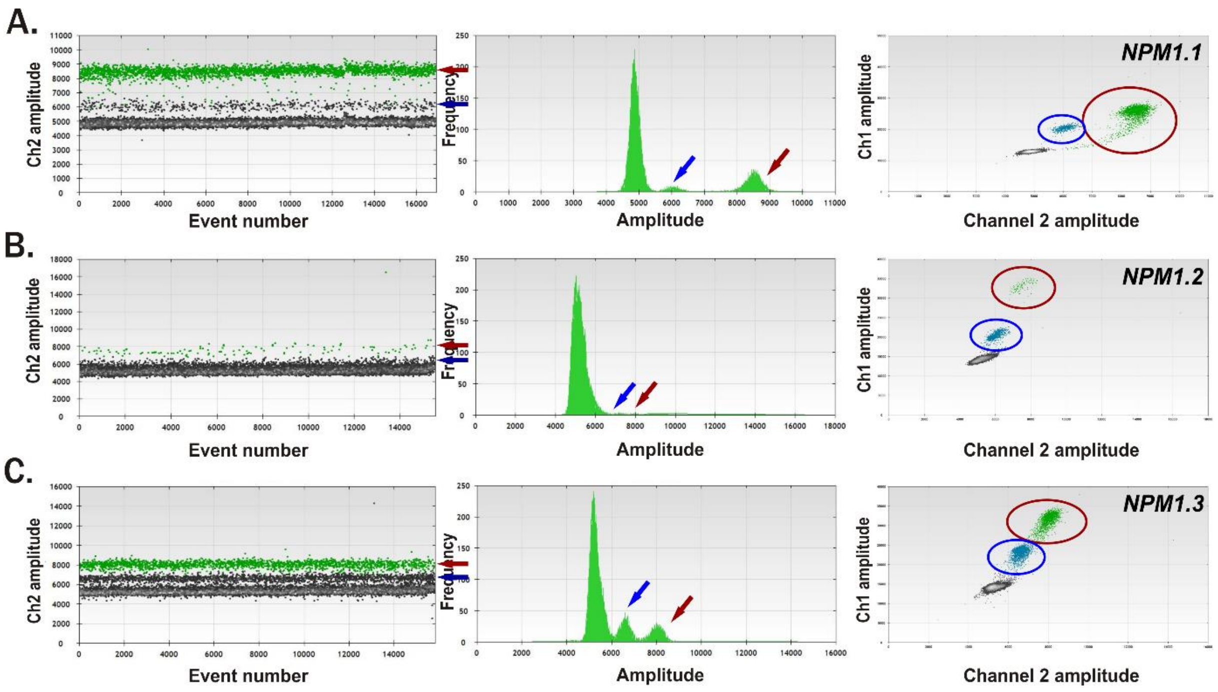

Figure S1 The graphic presentation of exemplary ddPCR results of expression analysis of *NPM1.1* (B), *NPM1.2* (C) and *NPM1.3* (D) transcripts. The plots were generated by Quanta Soft v. 1.5.38.1118 (Bio-Rad). The dark red arrows and circles indicate *NPM1* on a 1-dimensional plot (left panel), a histogram (middle panel) and a 2-dimensional plot (right panel). The dark blue arrows and circles indicate a reference gene *PGK1*. The groupings of grey dots below *PGK1* (left and right panels) and the highest peak on a histogram (middle panel) represent negative droplets, where no PCR product was detected due to the lack of a template.

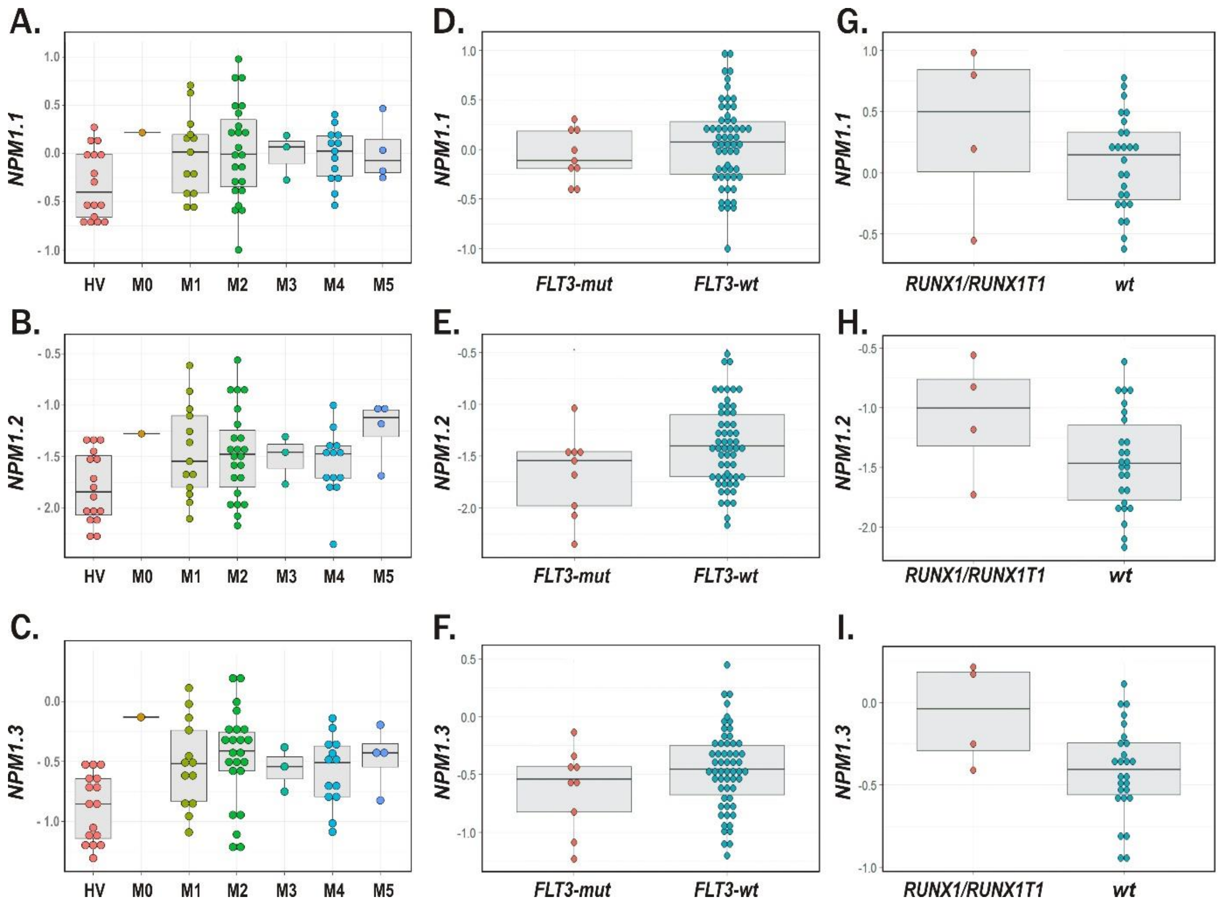

Figure S2 The comparison of three *NPM1* transcript levels in AML samples stratified by the FAB classification (A-C), *FLT3* mutation status (D-F), and translocation t(8;21) status, verified by the presence or absence of *RUNX1/RUNX1T1* fusion gene (G-I). Each dot represents one sample. Background boxplots show the median (a line in the middle) and the first and third quartiles (the bottom and top of the box). Due to the small number of samples per group statistical analysis was not applied. HV – healthy volunteers; M0-M5 – AML types according to the FAB (French-American-British) classification; *FLT3*-mut – samples with *FLT3*-ITD (internal tandem duplication); *FLT3*-wt – samples without *FLT3*-ITD (*FLT3* wild type); *RUNX1/RUNX1T1* – samples with t(8;21); wt – wild type samples (without t(8;21).

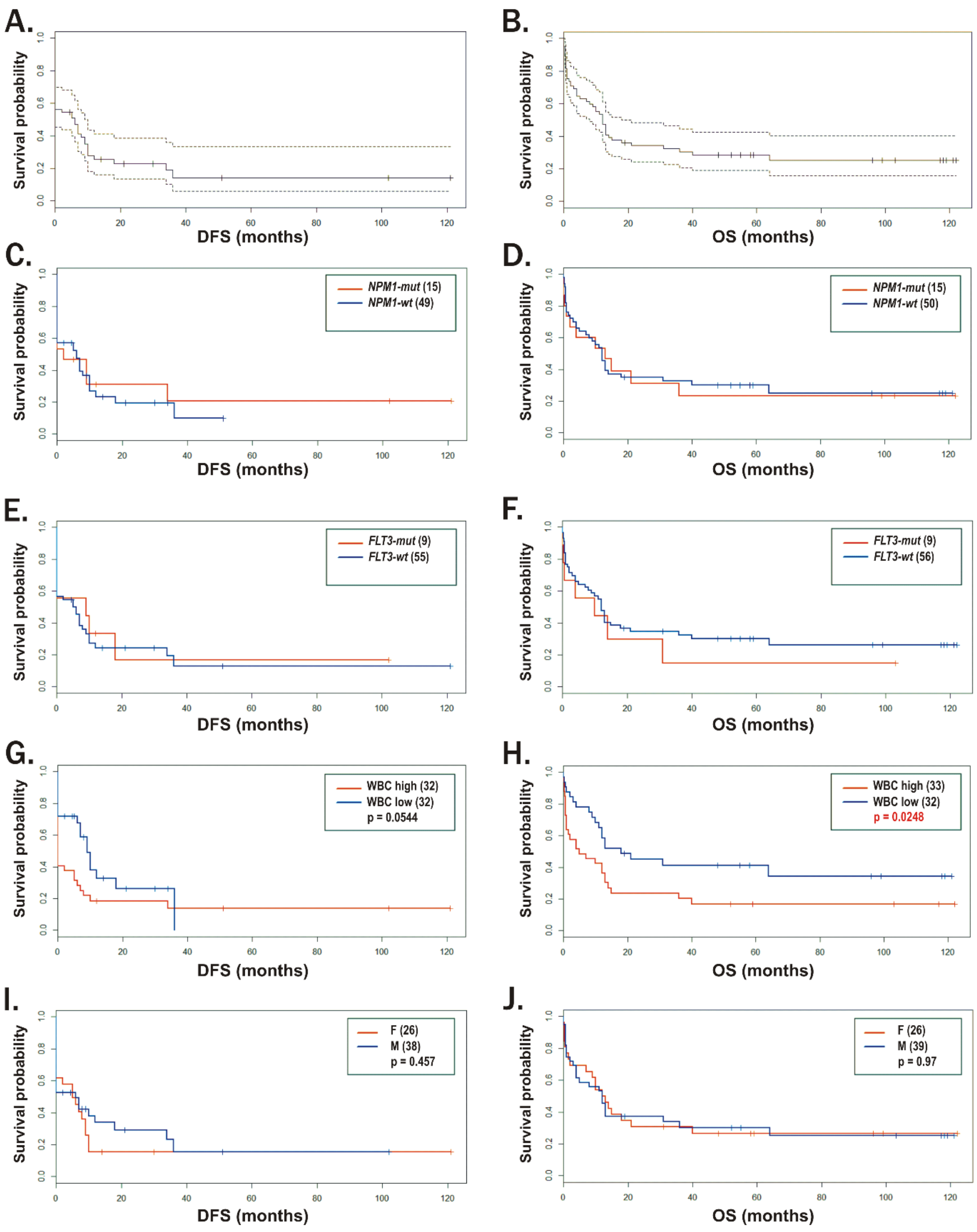

Figure S3 Disease free survival (DFS) and overall survival (OS) of 66 leukemia patients included into the study (A and B). DFS and OS of the same group of patients stratified by the *NPM1* mutation status (C and D), the *FLT3*-ITD mutation status (E and F), the WBC count (G and H), and sex (I and J). Due to the small number of samples with *FLT3*- and *NPM1*-mutation, statistical analysis was not applied in C-F.

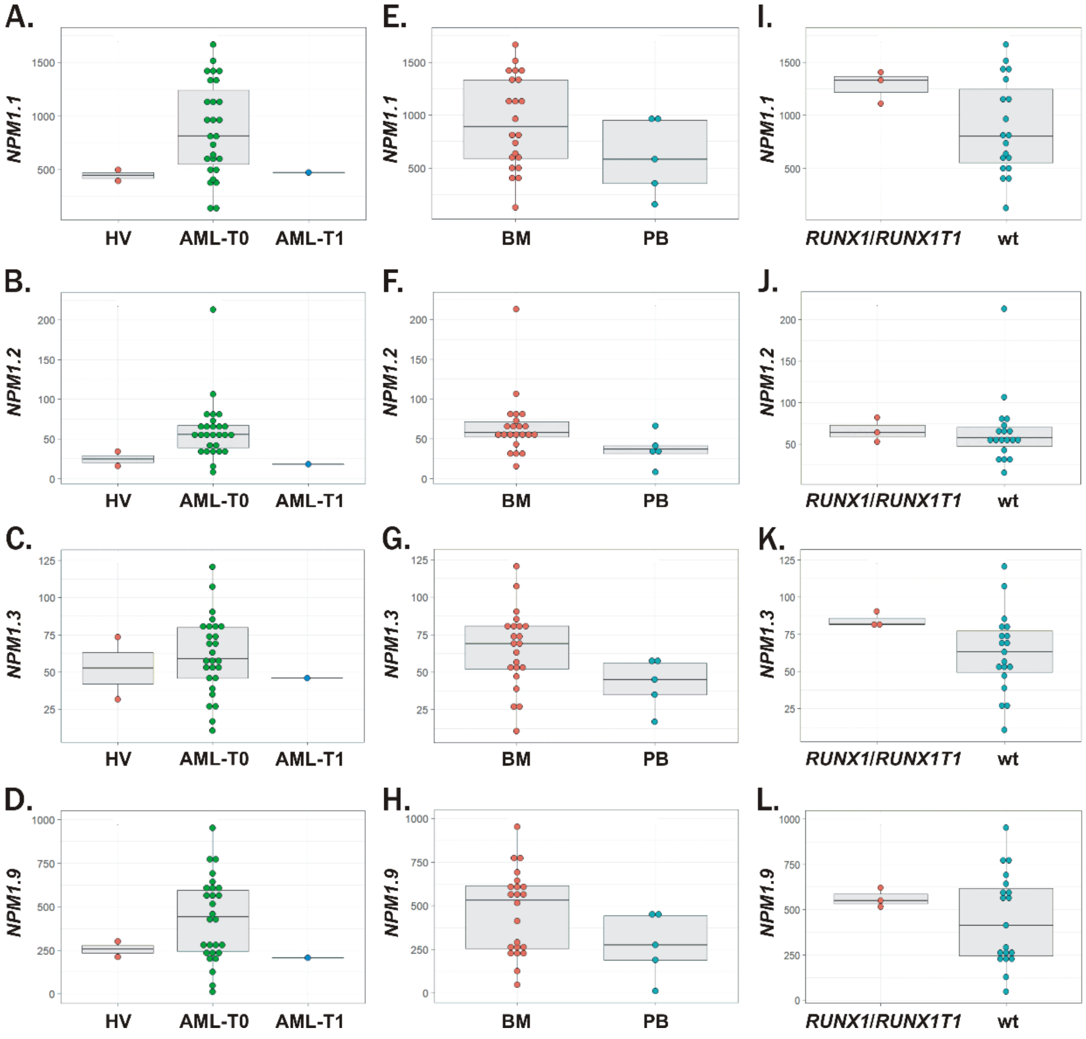

Figure S4 The RNA-seq-based comparison of the four most abundant *NPM1* transcript levels in AML-T0 samples compared to HV and AML-T1 (A-D), in AML-T0 samples stratified by the source tissue type (BM or PB) (E-H), and in AML-T0 samples with and without t(8;21) and the RUNX1/RUNX1T1 fusion gene. Analysis of t(8;21) impact was performed excluding PB samples. Each dot represents one sample. Background boxplots show the median (a line in the middle) and the first and third quartiles (the bottom and top of the box). Due to the small number of samples per group statistical analysis was not applied.

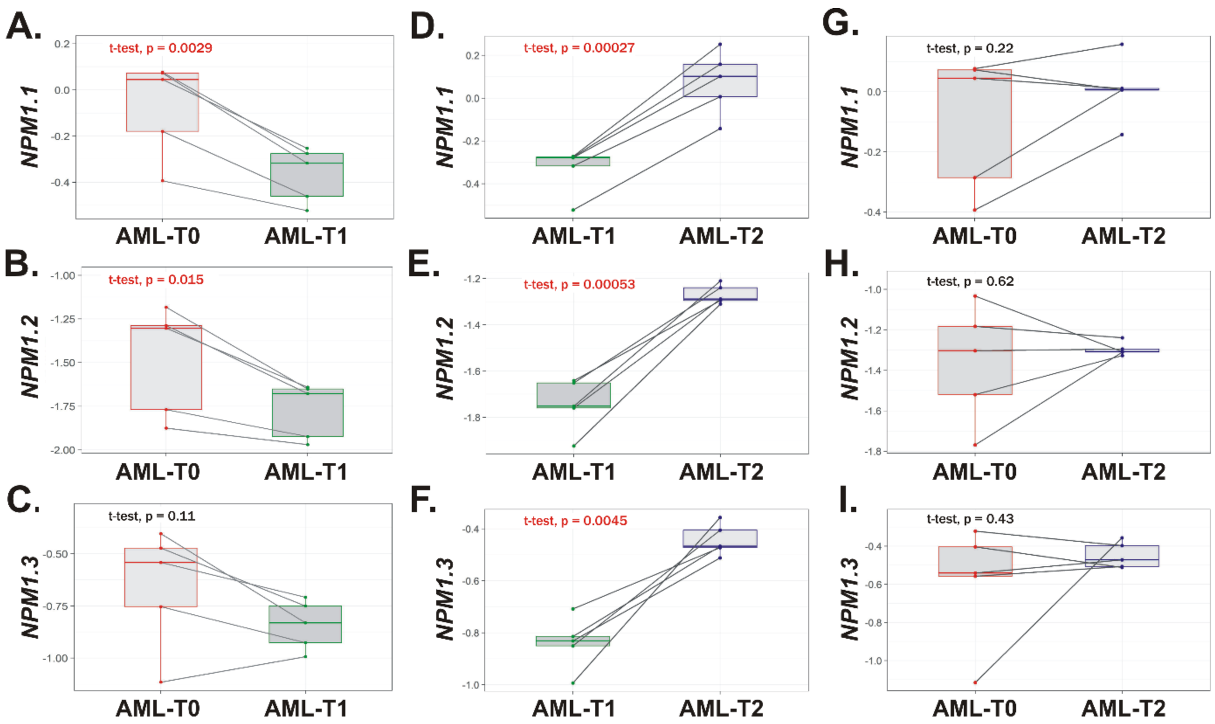

Figure S5 The pairwise comparisons of three *NPM1* transcript levels in AML patients samples collected at two time points: T0 (at the time of first diagnosis) and T1 (after treatment, in complete remission) (A-C); T1 and T2 (at the relapse) (D-F); T0 and T2 (G-I). Each dot represents one sample, lines connect the samples collected from the same patient. Background boxplots show the median (a line in the middle) and the first and third quartiles (the bottom and top of the box). Included paired t-test p values indicate the differences (statistically significant indicated in red). Plots A-C were drawn with the exclusion of two patients resistant to treatment.
